# Supplementary material for: The transcriptional landscape of a rewritten bacterial genome reveals control elements and genome design principles
Source: Nat Commun. 2021 May 24;12:3053. doi: 10.1038/s41467-021-23362-y (PMC8144410; doi:10.1038/s41467-021-23362-y)
Supplement: Supplementary file 1 — Supplementary Information [file 41467_2021_23362_MOESM1_ESM.pdf]

Supplementary Information

The transcriptional landscape of a rewritten bacterial genome reveals control elements and genome design principles

Mariëlle J.F.M. van Kooten[1]*, Clio A. Scheidegger[1], Matthias Christen[1] and Beat Christen[1]*

[1]Institute of Molecular Systems Biology, Department of Biology, Eidgenössische Technische Hochschule Zürich, CH-8093 Zürich, Switzerland

*Corresponding Authors: Mariëlle J.F.M. van Kooten, marielle.van.kooten@alumni.ethz.ch, Beat Christen, beat.christen@imsb.biol.ethz.ch

Supplementary Notes

Synonymous codon changes affect RNA stability

Dramatic changes in RNA stability can result from different biological mechanisms and affect the faithful expression of rewritten genes. To systematically assess alterations in RNA stability upon synonymous recoding, we examined the 103 CDS and non-coding genes in the category different coding region. Twelve genes showed a sharp de- and increase at unusual positions, namely in center portions of the genes. The sharp de- and increases at up to 95% of the maximum RCPB of the genes (Supplementary Data 2 Sheet 7) are indicative of degradation events at specific locations. Although the majority of bacterial endonucleases comes with scarce, if any, sequence specificity[1,2], the observed patterns resemble cleavage events as seen for RNase III in *E. coli*[3]. In the absence of a particularly striking sequence motif both in *E. coli*[3,4] and *C. crescentus*[5], RNase III has been suggested to recognize its target based on the double stranded structure[3]. To examine if we could attribute the effect seen in our dataset to RNase III cleavage events, we calculated the minimal free energy of 60 bp regions underlying the de- and increases (Supplementary Data 2 Sheet 7). Secondary structure stability was not affected upon recoding, whilst events were present in either the native or the rewritten counterpart, suggesting that the strong de- and increases were not solely due to RNase III activity and lie in the codon sequence, rather than the nucleotide sequence.

Translation speed can contribute to mRNA stability in bacteria[1]. A collection of features is thought to underlie slow translation, which includes Shine Dalgarno or SD-like motifs[6,7,8] and the time to encounter and decode tRNA, in which wobble pairing of the third base in translation[9,10], consecutive rare codons[11], homopolymeric codon runs[12], and specific codon pairs[13] slow the rate of translation. In the event of detrimental stalling, ribosome rescue induces mRNA cleavage near the arrest site[14,15,16,17], which would appear as a sequence-specific cleavage event in the mRNA profile. To examine the contribution of each of the features in more detail, first, we examined the sequences for the presence of SD-like motifs. Three CDS carry such motifs (CETH_00277, CETH_00899 and CETH_03090; Supplementary Data 2 Sheet 7). Whilst pairing of the 16S 3' anti-SD with SD-like motifs in the mRNA is thought to slow to ribosome[6,7], in these instances, the SD-like motifs are formed by recoded Glycine codons that are rare (GGT/A/G) and encoded for by a codon-specific isoacceptor (GGG) or an isoacceptor subject to wobble pairing (GGT). The former means that the tRNA with the anticodon is less abundant, leading on average to longer pausing of ribosomes. The latter leads to slower translation rates, as wobble base pairing is slower. To assess the effect of SD-like sequence motifs that involve recoded Glycine codons, we created a panel of translational fusions to *lacZ* of CCNA_00899 (128 aa) and CETH_00899 of increasing lengths (51 aa, 128 and 183 aa). The first construct does not include the SD-like motif GGA GGG (Gly116-Gly117), the second includes this sequence, and the third extends past the sequence. We measured the β-galactosidase activity of these constructs at 4'496 ± 138 (CCNA_00899), 4084 ± 95, 4'023 ± 32, and 3'618 ± 5 Miller Units (CETH_00899), respectively (Source Data). These results do not correspond to our initial observations based on RNA-Seq measurements, which show the instability at a strength of 92% (CETH_00899, Supplementary Data 2 Sheet 3). In our experimental setup, we noticed that the SD-like site in the second and third construct might function as an SD and is followed by a CTG start, which might explain why we cannot recapitulate our initial measurements, which would show low Miller Unit counts in precisely these two constructs as a result of mRNA cleavage. Consecutive rare codons with a codon-specific tRNA isoacceptor or isoacceptors that necessitate wobble pairing can explain the sharp de- and increases as observed in 4 additional sequences (CCNA_02635, CETH_02183, CETH_02975, two instances). To assess the effect of consecutive rare codons, we constructed two translational fusions to *lacZ*. Each of the constructs had, 14 aa upstream of *lacZ*, two tandems of rare codons at a one amino acid separation. The constructs either contained non-rare Gly-GGC, or rare (GGG) followed by rare and wobble paired (GGT) Gly-GGG/T. We measured the β-galactosidase activity of these constructs at 237 ± 4 and 188 ± 3 Miller Units, respectively (Source Data). Albeit the effect does not approach the up to 80% strength of the instability we observed in RNA-Seq measurements (CETH_02975, Supplementary Data 2 Sheet 3), given the limited (four-nucleotide) sequence difference between these constructs, these results do not necessitate the rejection of our initial hypothesis. Next, we examined the codon sequences of the remainder of genes and found 2 native genes to contain consecutive Pro codons (CCNA_00041 and CCNA_01651). Pro-Pro di-codons dramatically slow translation speed because of their uniquely bad behav-

ior as both donor and acceptor in peptide synthesis [13]. For 4 genes, we did not find specific sequence motifs that are known to affect translation speed, however, 2 of these genes are highly expressed and contain long stretches in the vicinity of the de- and increase with repetitive codons (e.g. 9 out of 10 codons code for Arg or Asp upstream of CCNA_00920 and 16 out of 20 codons code for Ala upstream of *exbB*) (Supplementary Data 2 Sheet 7). Consecutive codons may slow translation through depletion of the local tRNA concentration [12].

In summary, we do not expect a single mechanism to contribute to the changes in RNA stability that we observe for the recoded CDS. We were not able to fully recapitulate our initial observations that were based on RNA-Seq measurements in orthogonal measurements with, at the basis, translational fusions to *lacZ* of motifs that may render mRNA instability. In addition, there are examples of native and rewritten CDS that carry the elements we point out here, and yet do not display signs of detrimental ribosome stalling. We suggest to revise recoding in the appointed regions (Design Principle 5, Supplementary Table 4), although further investigation is necessary to determine the exact cause of the phenomenon. Measurements that assess the underlying mechanisms and contribute to an understanding of potential additive effects are out of the scope of this work. In essence, these measurements should recapitulate RNA-Seq observations and enable the measurement of longer sequences of 60 bp and up, should comprehensively incorporate the features individually and in a combinatorial manner, starting with the sequences of the 12 genes in Supplementary Data 2 Sheet 7. Finally, the biological function, if any, of the resultant motifs should be assessed. Here, the resultant protein is of importance too: slow translation can contribute to correct protein folding [18,19,20]. Taken together, the introduction of alternative synonymous mutations on a genome scale should allow to further untangle the contribution of transcriptional interference, nuclease-mediated degradation and translation to RNA stability, and as such make RNA stability a more controllable feature.

Supplementary Figures

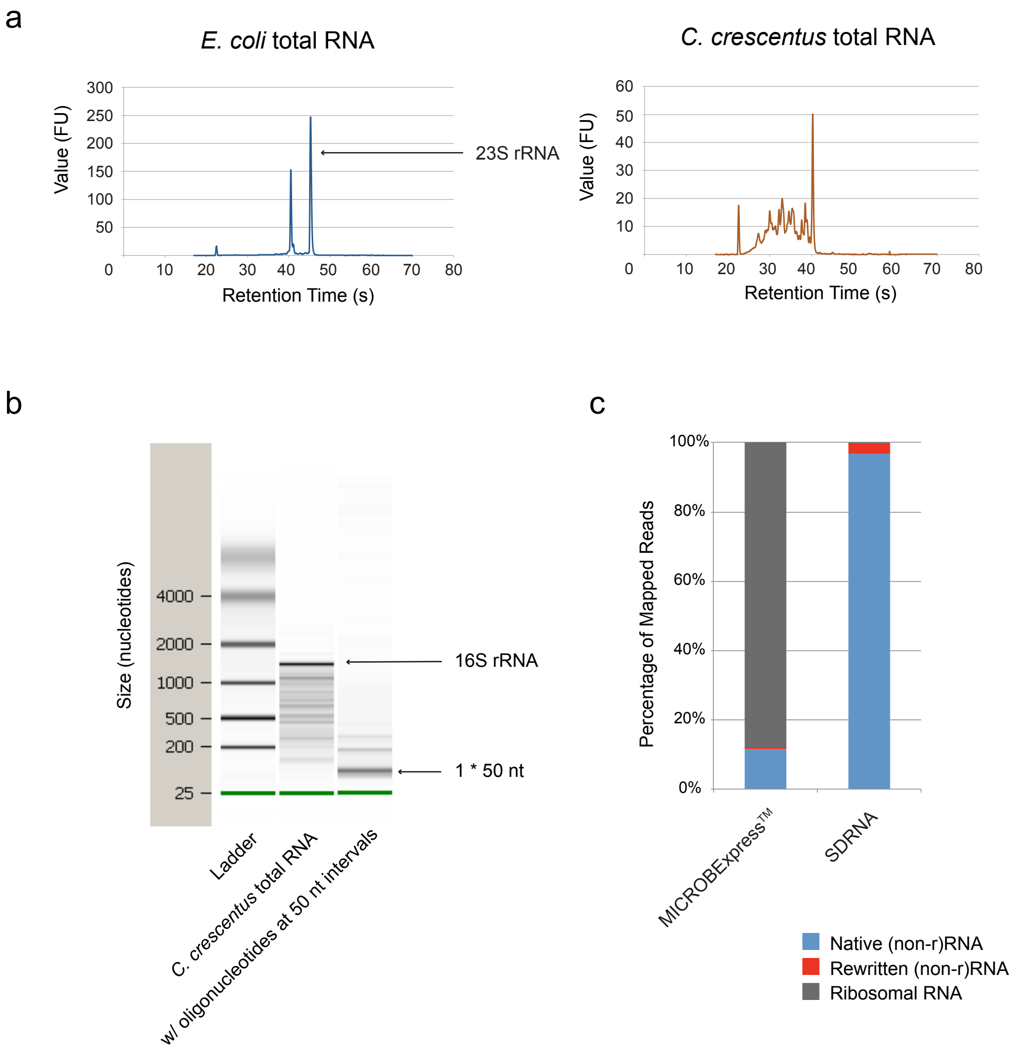

Supplementary Fig. 1: In-house selective depletion of ribosomal RNA (SDRNA). a. Unlike *Escherichia coli* 23S rRNA (left), *C. crescentus* 23S ribosomal RNA is processed into fragments (right). b. Complementary oligonucleotides and the digestion of RNA in RNA-DNA hybrids by RNase H is effective in the depletion of ribosomal RNA in *C. crescentus*, as shown by the addition of a first pool of oligonucleotides complementary to the ribosomal RNA, designed to anneal with the rRNA at intervals of 50 nucleotides. RNase H treatment and subsequent Bioanalyzer analysis showed RNA peaks starting at 50 nucleotides at regular intervals, suggesting that the bulk of RNA in the sample originates from degraded ribosomal RNA. Source data are provided as a Source Data file. c. A comparison of removal of ribosomal RNA through commercially available MICROBExpress™ and in-house selective depletion of RNA (SDRNA). Merosynthetic *C. crescentus* with, here, segment 9 and 10 have been subjected to removal of ribosomal RNA. Due to the fragmentation of 23S RNA the removal was incomplete when MICROBExpress™ is used, whereas the removal was complete upon SDRNA. The data depicted in Supplementary Figure 1 c and in Supplementary Table 1 have been derived from single RNA-Seq measurements for each procedure. All subsequent samples were depleted through SDRNA with similar results. Total counts and read counts per gene are given in Supplementary Data 1 - 3.

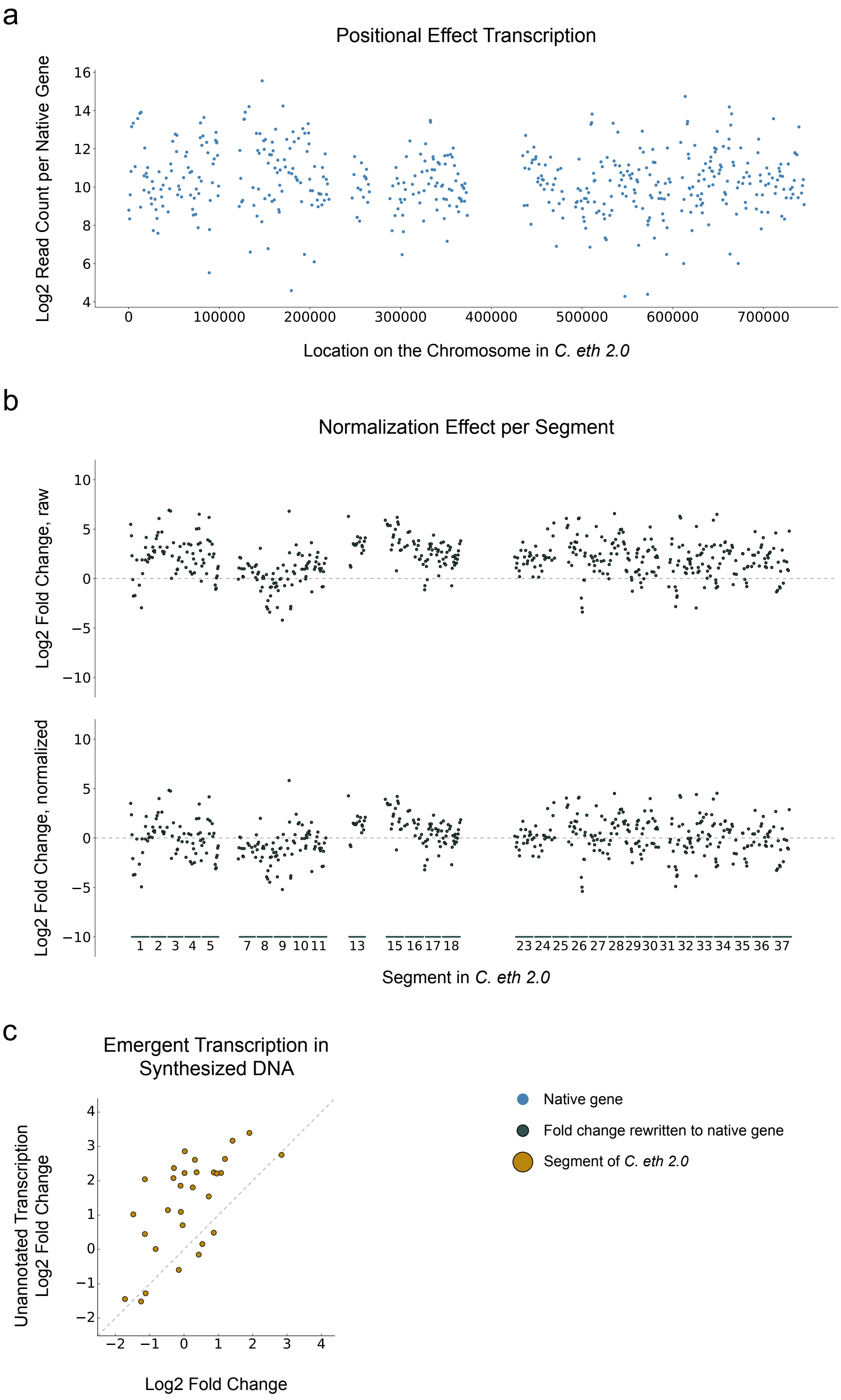

Supplementary Fig. 2: Strand-specific transcription from the native chromosome and plasmid-based, synthesized DNA in merosynthetic *Caulobacter crescentus*. a. The read count per gene (RCPG) of a native gene is not affected by the location on the chromosome. b. The rewritten RCPG has been normalized for plasmid copy number (Methods, Supplementary Data 1). c. A higher number of read counts mapped to locations not annotated as genes in constructs per segment of synthesized DNA when compared to the corresponding region in the native genome. An absence in apparent correlation with annotated transcription excludes generally higher levels of transcription as the cause of the phenomenon. This discrepancy was attributed to the omission of transcription termination elements and introduction of transcription start sites.

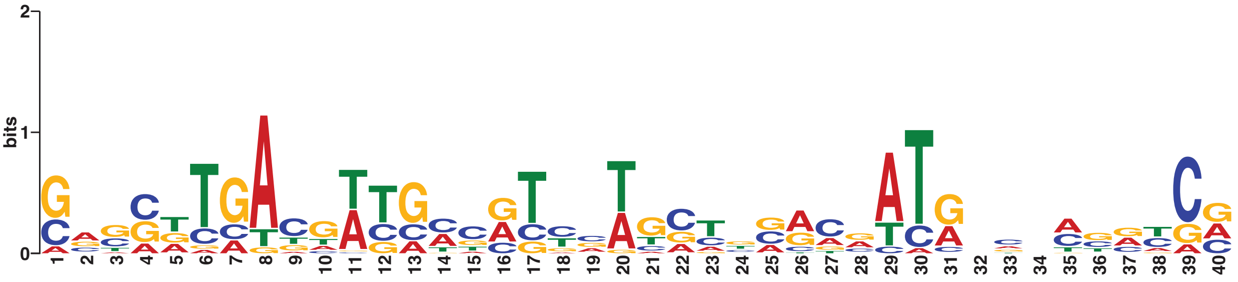

Supplementary Fig. 3: A MEME[21]-based pattern search in nucleotide sequences underlying emergent TSSs. One of two significant (E-value = 8.7e-010) potential motifs identified shows a pattern that resembles the -35 and -10 box RpoD consensus motif 5'-TTGaCgS-3' and 5'-GCtANAWC-3'[22], with 38 sites contributing to the construction of the motif. Two sites contributed to the construction of a second significant motif (E-value = 1.9e-003).

Annotation	Position	Read count	Maximum read count	Strand	Strength		-35		10 - 19	-10	
	481016	295	1184	-1	889	GAAACGATGC	TTGCCGC	CCAATG	AGCGCGCG	GCTACAAA	TCTAAG
							TGGCGGC			GCTACAAA	
antisense	179640	244	1147	1	903	GAAAAGACCG	TCGGCGC	GTCCGC	TTTCC	GCTAGGAC	CGCTGG
							TCGGCGC			GCTAGGAC	
antisense	26122	50	1029	1	979	CACCAGTTTT	TTGACCT	CCGCAA	GCGTTGGAGCT	GCTGCTTC	ACGTGC
							TTGACCT			GCGGCTTC	
antisense	292139	1651	7988	1	6337	TTGTAATGAT	TTGACGA	GCCGCT	TCCACTGT	GCTAATGC	CGCCAA
							CTGACGG			CGAGATCC	
antisense	346584	478	1967	1	1489	GAGTTTATCT	TTGTTGC	GCTGCT	CCCATTCTTGC	GTTAGGTC	GGGGAA
							TTGTTGC			GTCAGGTC	
antisense	457797	991	4017	1	3026	TAGCGCTAGG	TTGAGGC	TCCTCG	TTAT	GATAAATC	TGCAGG
							CTGCGGC			GATAAATC	
sense	91044	259	1086	1	827	CGTGCGAAAA	TTGAGGG	TGCGAG	TGTGCTTCTC	GCTAGCGC	CACCCC
							TCGAGGG			GCCTCGGC	

Supplementary Fig. 4: The RpoD -35 box and -10 box consensus motif underlie TSSs in the rewritten genome of *C. eth 2.0*. A comparison of the putative rewritten -35 and -10 box in the rewritten genome of *C. eth 2.0* (blue) to the nucleotide sequence at the corresponding position in the native genome of *C. crescentus*, in which point mutations have been highlighted (gray) suggests that a single point mutation can jump-start transcription. Position 179640 is indicative of a TSS that maps back to the promoter of CCNA_00897, a non-essential gene that has been omitted from the genome of *C. eth 2.0*. The -35 box of CCNA_00897 is thereby mapped at 250 bp of the start of CCNA_00897.

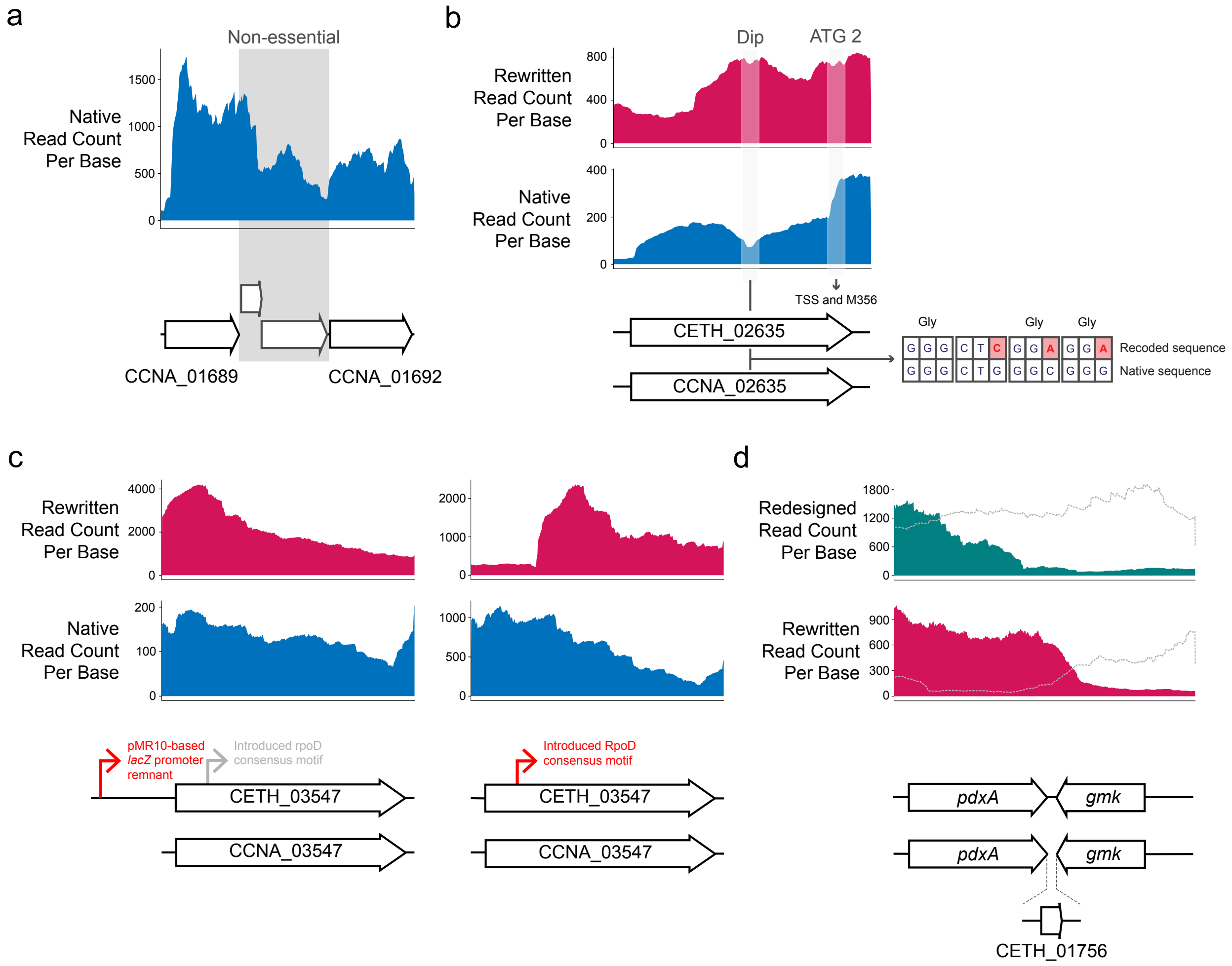

Supplementary Fig. 5: Detailed analysis of the transcript abundance along the genes encoding CMP synthase GuaA (CCNA_01692), FtsW (CCNA_02635) and LdpF (CCNA_03547). a. The sense-oriented, upstream gene CCNA_01691 of *guaA* (CCNA_01692) contains a sequence that terminates upstream transcription. CCNA_01691 was found to be non-essential and was omitted from the genome design. As a result, transcription from upstream CETH_01689 is not terminated leading to transcriptional read-through into CETH_01692. b. The transcription curves of CCNA_02635 and CETH_02635, which encode FtsW, show differences in the coding region. While a Shine Dalgarno-like motif thought to induce ribosome stalling [13,23,24] is found in the rewritten sequence, it is the *native* mRNA profile that shows a sharp de- and increase in the read count per base (RCPB) at this unusual position (inset). The sharp de- and increase in RCPB maps to 4 instances of codon GGG in a short (20 AA) codon stretch. In addition, *ftsW* has been reported to encode an mRNA isoform from a second ATG at position M356 with a putative RpoD binding site 35 bp upstream of ATG 2 [25]. Although recoding has not abolished either feature in CETH_02635, transcription from the second TSS is no longer observed. An RpoD consensus motif has been introduced in CETH_02635 upstream of the native RpoD consensus motif. c. CETH_03547 encoding peptidoglycan-specific endopeptidase LdpF as measured as a single segment (left) and in combination with segment 33, where segment 34 had been positioned downstream (right). We have introduced an RpoD promoter consensus motif in CETH_03547. When segment 33 was measured as a single segment (left), CETH_03547 is situated at the plasmid backbone-segment junction. The backbone contains a *lacZ* promoter remnant that fires into CETH_03547 and transcription initiation based on the introduced consensus motif is not observed. In the absence of the backbone-based promoter, the RpoD consensus motif serves as a TSS. d. Upon redesign, we removed CETH_01756 from the convergent center of *gmk* (CETH_01753) and *pdxA* (CETH_01758), placing *pdxA* and *gmk* in close vicinity. *gmk* transcription in the revised context appeared to affect *pdxA* transcripts. Blue trace: native transcription curve derived from native read count, magenta trace: rewritten transcription curve derived from rewritten read count, teal trace: redesigned transcription curve derived from redesigned read count, dashed line: antisense redesigned (top) or rewritten (bottom) read count.

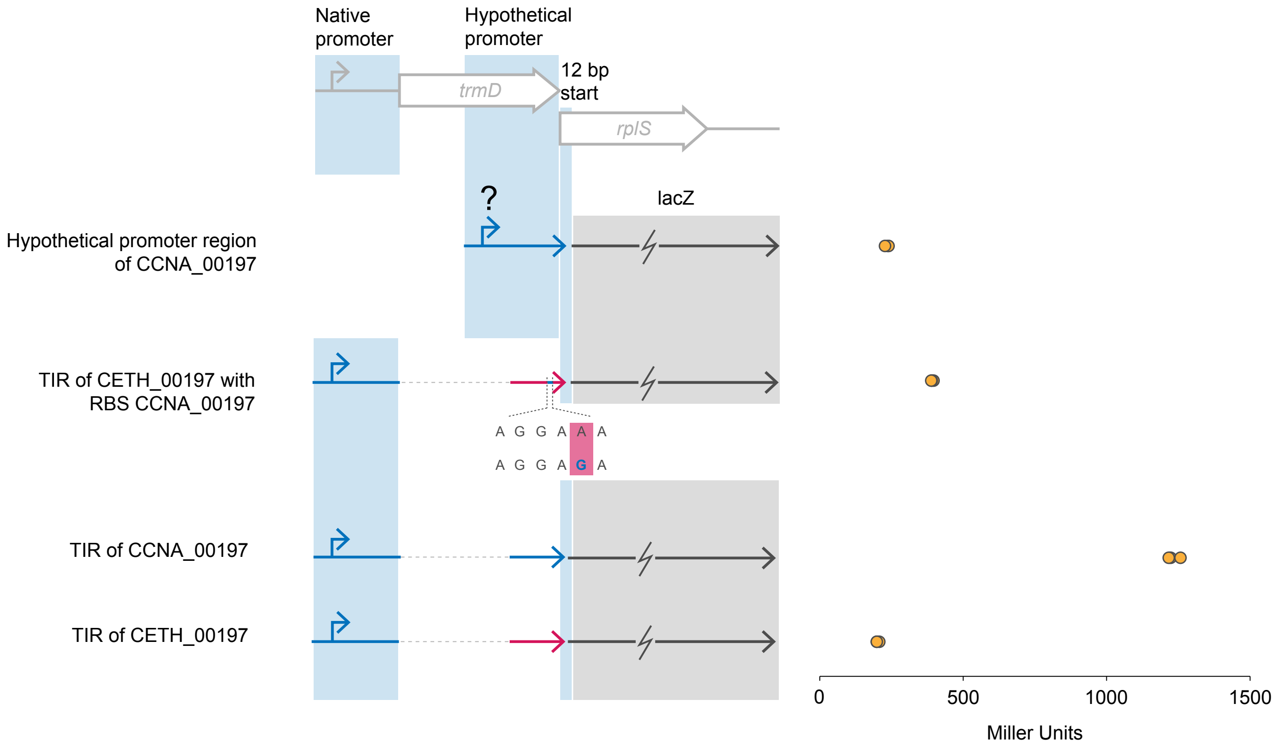

Supplementary Fig. 6: The coding region of the gene encoding tRNA methyltransferase TrmD facilitates translation of large subunit ribosomal protein L19, RplS as shown by translational fusions to β-galactosidase. In each construct, *lacZ* is proceeded by the 12 bp start of *rplS*, which is encoded by the same nucleotide sequences in the native and rewritten situation. To confirm the transcriptional coupling between *trmD* and *rplS*, we fused the hypothetical promoter region of *rplS* to *lacZ*. The promoter region of *trmD* gives rise to polycistronic mRNA, as we did not detect any substantial amount of β-galactosidase activity originating from this fusion construct (top). For the following constructs, we thus used the native promoter of *trmD*. Inclusion of the natural Shine Dalgarno sequence in the rewritten translation initiation region (TIR) of *rplS* (center) did not substantially increase LacZ activity. Fusion of the native or rewritten TIR of *rplS* to *lacZ* showed the TIR to contain an RNA-based level of control (bottom). Blue: native sequence, magenta: rewritten sequence. Data are presented as one dot (yellow) per independent measurement. Dots per sample amount to a total of n=3. Source data are provided as a Source Data file.

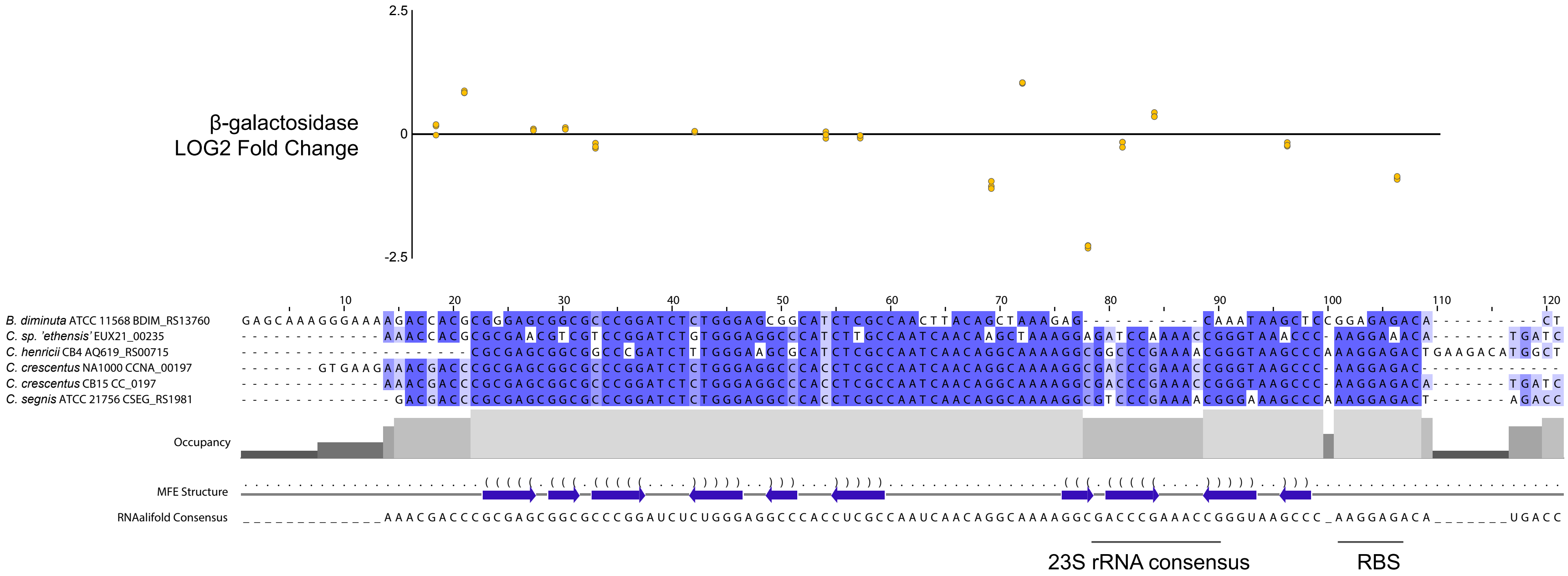

Supplementary Fig. 7: Based on *in silico* structural analysis and conservation analysis, the presence of translational control elements in *trmD* in the form of hairpin structures were predicted. To determine the contribution of individual recoded nucleotides to the decrease in translation of *rplS*, the 15 synonymous mutations introduced in the 100 bp upstream of the start codon of *rplS* were introduced in a panel of translational fusions to *lacZ* on a single-nucleotide, base-by-base level. The single nucleotide resolution revealed a regulatory hot spot (magenta) in the TIR of *trmD*. Data are presented as one dot (yellow) per independent measurement. Dots per sample amount to a total of n=3. Source data are provided as a Source Data file.

Supplementary Tables

Supplementary Table 1: A comparison of a commercially available kit and in-house SDRNA to deplete ribosomal RNA in *C. crescentus*.

**SAMtools**			**MICROBExpress™**	**SDRNA**
flagstat	QC-passed reads		59'103'963 (100%)	20'223'107 (100%)
idxstats	CCNA_R0066	23S rRNA	49'110'759 (83.09%)	5'728 (0.03%)
	CCNA_R0069	16S rRNA	2'696'385 (4.56%)	4'441 (0.02%)
	CCNA_R0067	tRNA-Ala	22'891 (0.04%)	53'521 (0.26%)
	CCNA_R0068	tRNA-Ile	22'928 (0.04%)	40'287 (0.20%)
	total rRNA		87.65%	0.05%

The data depicted in Supplementary Figure 1 c and in Supplementary Table 1 have been derived from single RNA-Seq measurements for each procedure. All subsequent samples were depleted through SDRNA with similar results. Total counts and read counts per gene are given in Supplementary Data 1 - 3.

Supplementary Table 2: Rho-dependent and independent transcription termination in non-essential genes of *Caulobacter crescentus*

	Gene Affected	Read Origin	Natural Termination	Element
Rho-independent	CETH_00137	CETH_00140	CCNA_00139	150935
	CETH_03793	CETH_03799	CCNA_03797	3960620
	CETH_01756	**CETH_01758**	CCNA_01757	1883516
	CETH_01633	CETH_01630	CCNA_01630	1750926
Rho-dependent	CETH_01692	CETH_01689	CCNA_01691	
	CETH_01629	CETH_01627	CCNA_01628	
	CETH_00309	CETH_00317	CCNA_00316	
	CETH_02210	CETH_02208	CCNA_02209	
	**CETH_00036**	CETH_00041	CCNA_00041	
	CETH_02333	CETH_02344	CCNA_02343	
	CETH_01809	**CETH_01803**	CCNA_01806	

Transcription termination elements as uncovered by transcription analysis of isolated essential and high-fitness parts. Values in the column Element refer to the start position of the hairpin structure from the 5' end. Genes in bold typeset have been repositioned upon redesign.

Supplementary Table 3: Putative control elements in the translation initiation regions of genes that show the same transcription curve but are faulty

Locus tag	Annotation	Genetic Context		Stability (kcal/mol) *	RBS	Start
		Coupled 5'	Coupled 3'			
CCNA_02623	cell division protein FtsZ	•		-15.69		
CCNA_01800	pyruvate dehydrogenase E1 component beta subunit	•			TGGAGGCCGCCGA	GTG
CCNA_01329	SSU ribosomal protein S11P	•				
CCNA_00763	electron transfer flavoprotein alpha subunit	•				
CCNA_02625	cell division protein FtsQ	•		-7.15	TGGAGGACGCTT	ATG
CCNA_00197	LSU ribosomal protein L19P	•		-13.24	AGGAGAC	ATG
CCNA_03913	hypothetical protein	•				
CCNA_01937	FeS assembly protein SufD	•			TGGGGGCGGCGGC	TTG
CCNA_02634	undecaprenyldiphospho-muramoylpentapeptide beta-N-acetylglucosaminyltransferase	•				
CCNA_02835	CpxP-related protein	•		-8.97	[illegible]GGACGCCCTTGAGGAGTCTTCCGCGCT	ATG
CCNA_01422	N utilization substance protein B NusB	•			TGGGGCAGGCCCG	ATG
CCNA_01222	NADH-ubiquinone oxidoreductase	•			TGGCCGGAACCT	ATG
CCNA_01803	pyruvate dehydrogenase complex, dihydrolipoamide acetyltransferase component	•				
CCNA_01328	SSU ribosomal protein S13P		•			
CCNA_00764	electron transfer flavoprotein beta subunit		•			
CCNA_03073	hypothetical protein		•			
CCNA_01792	LSU ribosomal protein L32P			-6.43		
CCNA_03934	hypothetical protein					
CCNA_01770	nucleoside diphosphate kinase					
CCNA_02631	P-loop NTPase-related protein					

*of detectable and altered secondary structure

The native and rewritten 100 bp region upstream of the start codon of 20 CDS. These regions have been altered as a result of a recoded and coupled upstream partner or the introduction of mutations to enable genome assembly. Translational regulation is predicted to occur based on transcription curve analysis and gene functionality measurements. The putative translational initiation region of the majority of these CDS (Coupled 5') lies in an upstream gene. Upon structural analysis, stability of the first secondary structure encountered upstream of the start codon was calculated. Together with SD recognition and conservation analysis (Methods), such analysis allowed to predict regions that should have been preserved. Marked nucleotides indicate synonymous mutations in the rewritten gene.

Supplementary Table 4: Principles for synthetic genome design

	Sequence elements involved	Observed (No.)	Validation	
			Orthogonal	Redesigned (No.)
Design Principle 1	Transcriptional regulatory elements	86		12
Design Principle 2	Transcription termination elements	18		4
Design Principle 3	RpoD consensus motif	76	Computational analysis<br>β-galactosidase assay	2
Design Principle 4	Translational regulatory elements	20	Computational analysis<br>β-galactosidase assay	2
Design Principle 5	Shine Dalgarno-like motifs, a.o.	13		

The presence of the sequence elements that underlie the principles was validated by computational analysis, which involved structural analysis, SD recognition and conservation analysis, or experimental analysis. Experimental analysis involved β-galactosidase assays, where single nucleotides were mutated in transcriptional or translational fusions, and RNA-Seq, where the redesign changes were implemented in synthesized DNA, and following RNA-Seq, transcription levels and transcription curves were analyzed.

Supplementary Table 5: Oligonucleotide table

Sequence	Construct
aggctactcgagcccttgagaaatacgccgtgcggtgtggcggaggggtgaatcaccgctttggccttgacgatgccatc	CETH_03009
gtggcaagctttacattatttacctcctccgcaggcggcgacgtggttatctttaatcaggatggcatcgtcaaggccaa	CETH_03009
aggctactcgagcccttgagaagtacgcggtgcgctgcggcggcggggtcaaccaccgctttggcctggatgacgccatc	CCNA_03009
gtggcaagctttgcattatttacctcctccgcaggccgcgacatggttgtccttgatcaggatggcgtcatccaggccaa	CCNA_03009
aggctactcgagcccttgagaaatacgccgtgcggtgtggcggaggggtgaatcaccgctttggcctcgacgatgccatc	CETH_03009 (repaired)
gtggcaagctttacattatttacctcctccgcaggcggcgacgtggttatctttaatcaggatggcatcgtcgaggccaa	CETH_03009 (repaired)
aggctactcgagcccttgtccgagcggaactggatcggctcgatcatgcgcgtatcgcggcgggagttgacgtggacgcg	CETH_03547
gtggcaagctttacattatttacctcctcgtgcattcagggcttcaagtctggcacgtttcgcgtccacgtcaactcccg	CETH_03547
aggctactcgagcccttgtgcgggccgagctggaccggctggaccatgcccggatcgccgccggcgtcgatgtcgacgcc	CCNA_03547
gtggcaagctttacattatttacctcctcgcgcgttcaaggcttcaagcctggcccgcttggcgtcgacatcgacgccgg	CCNA_03547
aggctactcgagcccttgtccgagcggaactggatcggctcgatcatgcgcgtatcgcggcgggagtggacgtggacgcg	CETH_03547 (repaired)
gtggcaagctttacattatttacctcctcgtgcattcagggcttcaagtctggcacgtttcgcgtccacgtccactcccg	CETH_03547 (repaired)
aggctactcgagcccttgttggccgaggtctgcttggtcgcgtggtgaatccacttggcgaacccattgacggcaaagga	CETH_03562
gtggcaagctttacattatttacctccttcaacccgccgacgctccgtgtactggatcggtcctttgccgtcaatgggtt	CETH_03562
aggctactcgagcccttgtcggccgtggcctgctgggccgcgtcgtcaacccgctgggcgagccgatcgacggcaagggc	CCNA_03562
gtggcaagctttacattatttacctccttcgacgcggcgacgctcggtgtactggatcgggcccttgccgtcgatcggct	CCNA_03562
aggctactcgagcccttgttggccgaggtctgcttggtcgcgtggtgaatccacttggcgaacccatagacggcaaagga	CETH_03562 (repaired)
gtggcaagctttacattatttacctccttcaacccgccgacgctccgtgtactggatcggtcctttgccgtctatgggtt	CETH_03562 (repaired)
aggctactcgagcccttg	universal PCA pcm
gtggcaagctttgcattatttacct	universal PCA pcm
aggctactcgagcccttgccgcagtacacgagaccg	CCNA_00197
gtggcaagctttggatgatgttgatcgccatgatcat	CCNA_00197
atcgataccggtggcgaattggagctccac	pPR9TT
cgatggcgcgccctcacgttaagggattttgg	pPR9TT
gaaacactggacattcgcgcct	CXXX_00198-CXXX_00197
gtaaaacgacgggatccccccttgatgatgttgatcgccatgatcat	CXXX_00198-CXXX_00197
agggaacaaaagctgggtac	prmCCNA_00198_CCNA_00198
ttcttgtgatcgcccgacag	prmCCNA_00198_CCNA_00198
tgctgtcgggcgatcacaagaagatcgaccaatggcgcaagc	universal GA TIR
gaccaatggcgcaagcgtatgcgtgaagaaacgacccgcgagcggcgcccggatctctgggaggcccacctcgccaatca	universal top
gttgatcgccatgatcatgtctccttgggcttacccggtttcgggtcgccttttgcctgttgattggcgaggtgggcctc	universal bottom
gaccaatggcgcaagcgtatgcgtgaagaaacCacccgcgagcggcgcccggatctctgggaggcccacctcgccaatca	1_C_univ_bot
gaccaatggcgcaagcgtatgcgtgaagaaacgacGcgcgagcggcgcccggatctctgggaggcccacctcgccaatca	2_G_univ_bot
gaccaatggcgcaagcgtatgcgtgaagaaacgacccgcgaAcggcgcccggatctctgggaggcccacctcgccaatca	3_A_univ_bot
gaccaatggcgcaagcgtatgcgtgaagaaacgacccgcgagcgTcgcccggatctctgggaggcccacctcgccaatca	4_T_univ_bot
gaccaatggcgcaagcgtatgcgtgaagaaacgacccgcgagcggcgTccggatctctgggaggcccacctcgccaatca	5_T_univ_bot
gaccaatggcgcaagcgtatgcgtgaagaaacgacccgcgagcggcgcccggatctGtgggaggcccacctcgccaatca	6_G_univ_bot
gaccaatggcgcaagcgtatgcgtgaagaaacgacccgcgagcggcgcccggatctctgggaggcccaTctcgccaatca	7_T_top
gttgatcgccatgatcatgtctccttgggcttacccggtttcgggtcgccttttgcctgttgattggcgagAtgggcctc	7_T_bot
gaccaatggcgcaagcgtatgcgtgaagaaacgacccgcgagcggcgcccggatctctgggaggcccacctTgccaatca	8_T_top
gttgatcgccatgatcatgtctccttgggcttacccggtttcgggtcgccttttgcctgttgattggcAaggtgggcctc	8_T_bot
gttgatcgccatgatcatgtctccttgggcttacccggtttcgggtcgccttttgcTtgttgattggcgaggtgggcctc	univ_top_9_A
gttgatcgccatgatcatgtctccttgggcttacccggtttcgggtcgcctttAgcctgttgattggcgaggtgggcctc	univ_top_10_T
gttgatcgccatgatcatgtctccttgggcttacccggtttcgggtcTccttttgcctgttgattggcgaggtgggcctc	univ_top_11_A
gttgatcgccatgatcatgtctccttgggcttacccggtttcggAtcgccttttgcctgttgattggcgaggtgggcctc	univ_top_12_T
gttgatcgccatgatcatgtctccttgggcttacccggtttTgggtcgccttttgcctgttgattggcgaggtgggcctc	univ_top_13_A
gttgatcgccatgatcatgtctccttgggTttacccggtttcgggtcgccttttgcctgttgattggcgaggtgggcctc	univ_top_14_A
gttgatcgccatgatcatgtTtccttgggcttacccggtttcgggtcgccttttgcctgttgattggcgaggtgggcctc	univ_top_15_A
gttgatcgccatgatcatgtctccttgggTttacccggtttcgggtcAccttttgcctgttgattggcgaggtgggcctc	univ_top_11_T_14_A
gttgatcgccatgatcatgtctccttgggcttacccggCttcgggtcgccttttgcctgttgattggcgaggtgggcctc	univ_top_add_G
gaccaatggcgcaagc	universal PCA TIR
gttgatcgccatgatcatgt	universal PCA TIR
ACGGGATCCCCCGGGCTGCAGAAGAGATCGCCGGCGAACA	CETH_00899
ACGGGATCCCCCGGGCTGCACAGCCGTTCGACGGCTTC	CETH_00899 - short
ACGGGATCCCCCGGGCTGCACACGCGTTGCACACGGTG	CETH_00899 - long
ACGGGATCCCCCGGGCTGCAGAAGAGGTCGCCGGCGAA	CCNA_00899
AAACTCCCCATGCGACTGCCCCAGGCCC	CCNA_00899
AGGCGGGCCTGGGGCAGTCGCATGGGGAGTTTCGCACG	GA CETH_00899 to CCNA_00899
CGAGGTCGACGGTATCGATACGGCGCCACATTCCCTTT	CETH_00899 - universal

Supplementary References

1. Hui, M. P., Foley, P. L. & Belasco, J. G. Messenger RNA degradation in bacterial cells. *Annual Review of Genetics* **48**, 537–559 (2014).

2. Bechhofer, D. H. & Deutscher, M. P. Bacterial ribonucleases and their roles in RNA metabolism. *Critical Reviews in Biochemistry and Molecular Biology* **54**, 242–300 (2019).

3. Gordon, G. C., Cameron, J. C. & Pfleger, B. F. RNA sequencing identifies new RNase III cleavage sites in Escherichia coli and reveals increased regulation of mRNA. *mBio* **8** (2017).

4. Pertzev, A. V. & Nicholson, A. W. Characterization of RNA sequence determinants and antideterminants of processing reactivity for a minimal substrate of Escherichia coli ribonuclease III. *Nucleic Acids Research* **34**, 3708–3721 (2006).

5. Bellofatto, V., Amemiya, K. & Shapiro, L. Purification and characterization of an RNA processing enzyme from Caulobacter crescentus. *Journal of Biological Chemistry* **258**, 5467–5476 (1983).

6. Li, G. W., Oh, E. & Weissman, J. S. The anti-Shine-Dalgarno sequence drives translational pausing and codon choice in bacteria. *Nature* **484**, 538–541 (2012).

7. Schrader, J. M. *et al.* The Coding and Noncoding Architecture of the Caulobacter crescentus Genome. *PLoS Genetics* **10** (2014).

8. Mohammad, F., Woolstenhulme, C. J., Green, R. & Buskirk, A. R. Clarifying the Translational Pausing Landscape in Bacteria by Ribosome Profiling. *Cell Reports* **14**, 686–694 (2016).

9. Crick, F. H. Codon—anticodon pairing: The wobble hypothesis. *Journal of Molecular Biology* **19**, 548–555 (1966).

10. Fluitt, A., Pienaar, E. & Viljoen, H. Ribosome kinetics and aa-tRNA competition determine rate and fidelity of peptide synthesis. *Computational Biology and Chemistry* **31**, 335–346 (2007).

11. Sørensen, M. A., Kurland, C. G. & Pedersen, S. Codon usage determines translation rate in Escherichia coli. *Journal of Molecular Biology* **207**, 365–377 (1989).

12. Zhang, G. *et al.* Global and local depletion of ternary complex limits translational elongation. *Nucleic Acids Research* **38**, 4778–4787 (2010).

13. Chevance, F. F., Le Guyon, S. & Hughes, K. T. The Effects of Codon Context on In Vivo Translation Speed. *PLoS Genetics* **10** (2014).

14. Roche, E. D. & Sauer, R. T. SsrA-mediated peptide tagging caused by rare codons and tRNA scarcity. *EMBO Journal* **18**, 4579–4589 (1999).

15. Hayes, C. S. & Sauer, R. T. Cleavage of the A site mRNA codon during ribosome pausing provides a mechanism for translational quality control. *Molecular Cell* **12**, 903–911 (2003).

16. Sunohara, T., Jojima, K., Tagami, H., Inada, T. & Aiba, H. Ribosome Stalling during Translation Elongation Induces Cleavage of mRNA Being Translated in Escherichia coli. *Journal of Biological Chemistry* **279**, 15368–15375 (2004).

17. Li, X., Hirano, R., Tagami, H. & Aiba, H. Protein tagging at rare codons is caused by tmRNA action at the 3′ end of nonstop mRNA generated in response to ribosome stalling. *Rna* **12**, 248–255 (2006).

18. Zhang, G., Hubalewska, M. & Ignatova, Z. Transient ribosomal attenuation coordinates protein synthesis and co-translational folding. *Nature Structural and Molecular Biology* **16**, 274–280 (2009).

19. Pechmann, S. & Frydman, J. Evolutionary conservation of codon optimality reveals hidden signatures of cotranslational folding. *Nature Structural and Molecular Biology* **20**, 237–243 (2013).

20. Jacobs, W. M. & Shakhnovich, E. I. Evidence of evolutionary selection for cotranslational folding. *Proceedings of the National Academy of Sciences of the United States of America* **114**, 11434–11439 (2017).

21. Bailey, T. L. & Elkan, C. Fitting a mixture model by expectation maximization to discover motifs in biopolymers. *Proceedings / ... International Conference on Intelligent Systems for Molecular Biology ; ISMB. International Conference on Intelligent Systems for Molecular Biology* **2**, 28–36 (1994).

22. Malakooti, J., Shui Ping Wang & Ely, B. A consensus promoter sequence for Caulobacter crescentus genes involved in biosynthetic and housekeeping functions. *Journal of Bacteriology* **177**, 4372–4376 (1995).

23. Li, G. W., Oh, E. & Weissman, J. S. The anti-Shine-Dalgarno sequence drives translational pausing and codon choice in bacteria. *Nature* **484**, 538–541 (2012).

24. Mohammad, F., Woolstenhulme, C. J., Green, R. & Buskirk, A. R. Clarifying the Translational Pausing Landscape in Bacteria by Ribosome Profiling. *Cell Reports* **14**, 686–694 (2016).

25. Schrader, J. M. *et al.* The Coding and Noncoding Architecture of the Caulobacter crescentus Genome. *PLoS Genetics* **10** (2014).
