# Supplementary material for: The transcriptional landscape of a rewritten bacterial genome reveals control elements and genome design principles
Source: Nat Commun. 2021 May 24;12:3053. doi: 10.1038/s41467-021-23362-y (PMC8144410; doi:10.1038/s41467-021-23362-y)
Supplement: Supplementary file 3 — Description of Additional Supplementary Files [file 41467_2021_23362_MOESM3_ESM.pdf]

Title: Supplementary Data 1
Description: contains metadata, the data normalization procedure, and the separation procedure for pooled strains.

Title: Supplementary Data 2
Description: contains processed and analyzed data for rewritten genes.

Title: Supplementary Data 3
Description: contains processed and analyzed data for redesigned genes.

Title: Supplementary Data 4
Description: lists the gBlocks that have been designed and used in this work.

Title: Supplementary Data 5
Description: lists strains used and created in this work.
