# Supplementary material for: The transcriptional landscape of a rewritten bacterial genome reveals control elements and genome design principles
Source: Nat Commun. 2021 May 24;12:3053. doi: 10.1038/s41467-021-23362-y (PMC8144410; doi:10.1038/s41467-021-23362-y)
Supplement: Supplementary file 9 — Reporting Summary [file 41467_2021_23362_MOESM9_ESM.pdf]

nature research

Corresponding author(s): Mariëlle J.F.M. van Kooten and Beat Christen

Last updated by author(s): May 10, 2021

Reporting Summary

Nature Research wishes to improve the reproducibility of the work that we publish. This form provides structure for consistency and transparency in reporting. For further information on Nature Research policies, see Authors & Referees and the Editorial Policy Checklist.

Statistics

For all statistical analyses, confirm that the following items are present in the figure legend, table legend, main text, or Methods section.

n/a	Confirmed	
☐	☒	The exact sample size (*n*) for each experimental group/condition, given as a discrete number and unit of measurement
☒	☐	A statement on whether measurements were taken from distinct samples or whether the same sample was measured repeatedly
☐	☒	The statistical test(s) used AND whether they are one- or two-sided<br>*Only common tests should be described solely by name; describe more complex techniques in the Methods section.*
☒	☐	A description of all covariates tested
☒	☐	A description of any assumptions or corrections, such as tests of normality and adjustment for multiple comparisons
☐	☒	A full description of the statistical parameters including central tendency (e.g. means) or other basic estimates (e.g. regression coefficient) AND variation (e.g. standard deviation) or associated estimates of uncertainty (e.g. confidence intervals)
☐	☒	For null hypothesis testing, the test statistic (e.g. *F*, *t*, *r*) with confidence intervals, effect sizes, degrees of freedom and *P* value noted<br>*Give P values as exact values whenever suitable.*
☒	☐	For Bayesian analysis, information on the choice of priors and Markov chain Monte Carlo settings
☒	☐	For hierarchical and complex designs, identification of the appropriate level for tests and full reporting of outcomes
☐	☒	Estimates of effect sizes (e.g. Cohen's *d*, Pearson's *r*), indicating how they were calculated

Our web collection on statistics for biologists contains articles on many of the points above.

Software and code

Policy information about availability of computer code

Data collection	Strand-specific, paired-end NGS of RNA was performed (GATC Biotech at Eurofins Genomics) on a HiSeq 4000 or NovaSeq 6000 S2 (Illumina).
Data analysis	Snakemake, bash and Python were used throughout data processing and analysis. To process raw data to RCPG and RCPB, Trimmomatic 0.36, BWA-MEM 0.7.12, SAMtools, HTSeq 0.9.1 and BEDTools, and R and DESeq2 were used. For data analysis, BLASTN 2.11.0+, MEME and FIMO 5.2.0, and RNAfold and RNAeval 2.4.11 were used. To display results, the Python library DNA Features Viewer, MUSCLE 3.8 and Jalview were used. All software used in this study is also denoted in the sections Results and Methods. Where version numbers are not provided, the authors used several versions throughout data processing and analysis and do not expect discrepancies in results between different versions. The code to process and analyze RNA-Seq data derived from merosynthetic strains is available from M.v.K. upon reasonable request.

For manuscripts utilizing custom algorithms or software that are central to the research but not yet described in published literature, software must be made available to editors/reviewers. We strongly encourage code deposition in a community repository (e.g. GitHub). See the Nature Research guidelines for submitting code & software for further information.

Data

Policy information about availability of data

All manuscripts must include a data availability statement. This statement should provide the following information, where applicable:
- Accession codes, unique identifiers, or web links for publicly available datasets
- A list of figures that have associated raw data
- A description of any restrictions on data availability

The RNA-Seq data has been submitted to the NCBI Sequence Read Archive under BioProject ID PRJNA695449 (https://www.ncbi.nlm.nih.gov/bioproject/PRJNA695449). Supplementary Data 1 lists metadata, the data normalization procedure and the separation procedure for pooled strains. Supplementary Data 2 and 3 list read counts per gene. The gBlock Table lists the gBlocks that have been designed and used in this work. The Strain Table lists strains used and created in this

work. Source Data are provided with this paper. The Source Data contain the measurement values for the β-galactosidase measurements that have been performed. Supplemental data that support the findings of this study are available from the corresponding authors upon reasonable request.

Field-specific reporting

Please select the one below that is the best fit for your research. If you are not sure, read the appropriate sections before making your selection.

☒ Life sciences ☐ Behavioural & social sciences ☐ Ecological, evolutionary & environmental sciences

For a reference copy of the document with all sections, see nature.com/documents/nr-reporting-summary-flat.pdf

Life sciences study design

All studies must disclose on these points even when the disclosure is negative.

Sample size	NGS of RNA was performed on RNA extracted from bulk cell culture. Each RNA sample contained the RNA of at minimum 1.5 ml of cell culture at an OD600 of at minimum 0.1.
Data exclusions	No data was excluded. Gene exclusion took place based on predefined principles and as stated in the section Methods, Gene exclusion principles. First, 2 genes where an unlabeled copy was present were discarded (CETH_R0045 and CETH_R0080, 768 - 4 = 764). Next, genes that do not match in length (764 - 23 = 741), that have not been recoded (741 - 52 = 689), that have been denoted as present in regions that have been deleted in vivo (689 - 64 = 625) (Venetz et al., 2019) and that are not fully contained on a single segment (625 - 13 = 612) were discarded. In addition to the denoted exclusions, for the analysis of isolated parts, genes (612 - 106 = 506) contained on segments that showed aberrant behaviour upon PCN correction were excluded (Supplementary Fig. 2 b). For the analysis of recoded parts, prior to transcription curve comparison, genes were discarded where the native and / or rewritten RCPG was below 16 (612 - 11 = 601) after depooling and application of plasmid copy number correction. The aberrant behaviour upon PCN correction that leads to the exclusion of genes in transcription level comparison does not hinder transcription curve comparison.
Replication	The β-galactosidase activities reported in the main text represent the average of at least three independent measurements derived from mid-log phase cultures. The β-galactosidase activities reported in the Supplementary Notes represent the average of at least two independent measurements derived from mid-log phase cultures. All attempts at replication, both of rRNA depletion by commercial methods and by in-house SDRNA, as well as of β-galactosidase measurements, were successful, i.e. the results supported the findings as presented.
Randomization	Merosynthetic strains or the RNA extracted from merosynthetic strains were pooled prior to rRNA depletion. Pooling was done without a predefined scheme. Strains cultured for β-galactosidase measurements were inoculated without a predefined scheme and samples were measured once mid-log phase had been reached.
Blinding	With respect to pooled merosynthetic strains, extracted and depleted RNA and raw data, the researcher was blinded and samples were processed as such. Here, blinding had a logistical purpose rather than an experimental purpose. For the measurement of β-galactosidase activities, the researcher was blinded: strains cultured for β-galactosidase measurements were labeled with their corresponding strain numbers. The plasmid content of these strains was assessed only upon data analysis.

Reporting for specific materials, systems and methods

We require information from authors about some types of materials, experimental systems and methods used in many studies. Here, indicate whether each material, system or method listed is relevant to your study. If you are not sure if a list item applies to your research, read the appropriate section before selecting a response.

Materials & experimental systems

n/a	Involved in the study	
☒	☐	Antibodies
☒	☐	Eukaryotic cell lines
☒	☐	Palaeontology
☒	☐	Animals and other organisms
☒	☐	Human research participants
☒	☐	Clinical data

Methods

n/a	Involved in the study	
☒	☐	ChIP-seq
☒	☐	Flow cytometry
☒	☐	MRI-based neuroimaging
